# Examining the experiences of pediatric mental health care providers during the early stage of the COVID-19 pandemic

**DOI:** 10.1186/s40359-023-01170-x

**Published:** 2023-05-04

**Authors:** Katherine Bright, Emma Cullen, Olivia Conlon, Rosslynn T. Zulla, David B. Nicholas, Gina Dimitropoulos

**Affiliations:** 1 Faculty of Nursing, University of Calgary, Calgary, AB Canada; 2 Department of Community Health Sciences, Cumming School of Medicine, University of Calgary, Calgary, AB Canada; 3 The Mathison Centre for Mental Health Research & Education, Cumming School of Medicine, University of Calgary, Calgary, AB Canada; 4 Faculty of Social Work, University of Calgary, Central and Northern Alberta Region, Edmonton, AB Canada; 5 Faculty of Social Work, University of Calgary, 2500 University Dr NW MacKimmie Tower (MT) 301, Calgary, AB T2N 1N4 Canada

**Keywords:** COVID 19, Qualitative research, Mental Health, Mental Health Providers

## Abstract

**Background:**

The COVID-19 pandemic fundamentally impacted the way that mental health services were provided. In order to prevent the spread of infection, many new public health precautions, including mandated use of masks, quarantine and isolation, and closures of many in-person activities, were implemented. Public health mandates made it necessary for mental health services to immediately shift their mode of delivery, creating increased confusion and stress for mental health providers. The objective of this study is to understand the impact of pandemics on the clinical and personal lives of mental health providers working with children during the early months of the COVID-19 pandemic, March -June 2020.

**Methods:**

Mental health providers (n = 98) were recruited using purposive sampling from a public health service in Canada. Using qualitative methods, semi-structured focus groups were conducted to understand the experiences of mental health service providers during the beginning of the COVID-19 pandemic.

**Results:**

Data from the focus groups were analysed and three main themes emerged: (1) shift to virtual delivery and working from home; (2) concerns about working in person; (3) exhaustion and stress from working through the pandemic.

**Discussion:**

This study gave voice to mental health providers as they provided continuity of care throughout the uncertain early months of the pandemic. The results provide insight into the impact times of crisis have on mental health providers, as well as provide practical considerations for the future in terms of supervision and feedback mechanisms to validate experiences.

**Supplementary Information:**

The online version contains supplementary material available at 10.1186/s40359-023-01170-x.

## Background

The novel coronavirus (COVID-19) was first identified in December 2019 in Wuhan, China [1] and declared a global pandemic by the World Health Organization on March 11, 2020 [1]. COVID-19 spread rapidly, passing between individuals in close contact [2, 3]; it quickly travelled the globe, reaching one million worldwide cases by April 1, 2020, and doubling only nine days after [4]. In Canada, the first case identified was reported on January 25, 2020, and within 45 days, case numbers had reached just under 100 [5]. The most common symptoms of the virus were reported as fever and dry cough [3], and are similar in presentation to previous respiratory viruses, such as Severe Acute Respiratory Syndrome (SARS) and Middle East Respiratory Syndrome (MERS) [3]. While the fatality of the disease is less than these other recent pandemics, COVID-19 spread rapidly leading to the healthcare systems being overburdened [6, 7]. In attempts to prevent the healthcare system from being overwhelmed, many public health measures were implemented, including quarantine and isolation, closures of schools and other gathering sites, work-from-home orders [1, 4, 6, 8–10] Despite efforts to curb the spread of the virus, health care systems became overwhelmed placing a large burden on frontline health care providers.

Following the declaration of the pandemic, frontline physicians, nurses and various other healthcare providers have been experiencing unprecedented challenges and frequent changes to public health policies and procedures [8]. Many changes were implemented rapidly throughout the pandemic and were designed to better serve the community and ensure continuity of care to young people and families. Health care providers, mainly those offering medical care, continued to provide necessary services to patients during the pandemic and many experienced high degrees of stress, anxiety and fear, as they navigated how to continue delivering adequate services while keeping community members safe, while quickly shifting to a new working environment [8, 11, 12]. In research conducted with psychotherapists who primarily worked with adult patients, many noted that the sudden shift to teletherapy came with many difficulties, and emphasized that those with previous telehealth experience had an easier time with delivery this type of care [12].

In past pandemics, such as SARS and H1N1, health care providers experienced increased levels of anxiety and other symptoms of mental health concern [13–15]. Pediatric health care workers felt increased pressure when working through the SARS pandemic, with many noting increased levels of emotional stress, including loneliness, sadness and fear [16]. Some of the heightened levels of anxiety and stress were connected to the task of working through the pandemic and concern about contracting or spreading the virus, particularly noted amongst research conducted with front line medical staff such as physicians and nurses [13, 14].

A rapid systematic review conducted in 2020, following the outbreak of COVID-19, confirmed increasing incidences of depression and anxiety amongst the medical staff working through the pandemic, noting that health care workers, the majority of whom were identified as doctors and nurses, reported increased levels of anxiety, depression and insomnia [7]. It has been further noted that many health care workers take on additional stress from their clients. In a study conducted with 339 therapists working with adult patients experiencing trauma during the COVID-19 pandemic, almost 75% of participants reported having felt drained and nearly half of participants had a difficult time connecting with clients [17]. As a result, it was reported that participants experienced a moderate level of vicarious trauma while delivering care during the COVID-19 pandemic [17]. Previous studies have also found the health care providers, working during a pandemic, are more likely to report symptoms of burnout and post-traumatic stress [7, 18]. Healthcare workers noted that they felt concern about working through the pandemic, a concern that has been noted in previous pandemics as well. A qualitative study of 17 internal medicine residents working in Toronto during the previous SARS outbreak discussed the stress and anxiety medical staff have when caring for those infected with a highly transmissible disease while also being concerned about their own personal health and safety in the unfamiliar terrain of a pandemic [19].

Surveys with adult-serving psychotherapists noted that many felt a diminishing sense of competence in their ability to work with their clients [17]. The same psychotherapists further highlighted that they felt more tired following sessions with their clients during the pandemic [17]. Qualitative interviews conducted with 19 therapists, primarily working with an adult population, found that therapists struggled to support clients with trauma, as they were experiencing their own trauma during the COVID-19 pandemic [12]. In a review paper collating experiences of mental health providers during the COVID-19 pandemic, it was noted that as the pandemic went on, there was a worsening of clinician mental health, validating that the working conditions experienced during the pandemic had a negative impact on healthcare clinicians [7]. In a pediatric clinicians’ online roundtable discussion [20], it emerged that the mental health providers themselves were struggling with personal challenges related to the pandemic; many were caring for their own children or aging parents. Some were worried about their own or family members’ health if they were required to be in hospital or work face-to-face with patients. However, in the midst of all these, mental health professionals (MHPs) organically formed online support groups not only for consultation for work-related stress but also to support each other emotionally. This demonstrates mental health challenges in caregiver populations and the importance of employers considering the mental well-being of their employees.

With the onset of the pandemic, many front-line medical professionals, including Emergency Medical Technicians (EMT), physicians, nurses and clinical managers, experienced an increased volume of clients, overwhelming workloads and more acute cases [11, 21]. Health care clinicians found that the collective weight of these stressors impacted both their work and home lives, however clinicians continued to provide care for their clients under increasingly stressful conditions [11]. Lack of preparation in providing care during a pandemic has resulted in an increase in stress for health care workers, leading to more potential for burnout, as highlighted in a study of Ghanese doctors, nurses and allied health workers [22]. For some healthcare providers, the difficulty in providing adequate care to clients during the COVID-19 pandemic contributed to employee burnout and desire to leave the job or profession [11].

In one study, healthcare workers in Ontario, Canada, were infected more than the general public. In-depth interviews with ten frontline healthcare workers reported anxiety related to the potential of infecting family members. Increased workloads, exhaustion and burnout were also reported. Participants reported a sense of abandonment from government and leadership, noting inadequate access to personal protective equipment (PPE), inconsistent and rapidly changing policies and protocols, and a lack of initial planning for this pandemic [23].

A survey of mental health therapists, primarily working in private practice with an adult population, found they had little time to prepare for the shift in delivery of services, with little guidance and difficulty in accessing patient files and confusion around procedures [12]. A difficulty that many clinicians experienced throughout the pandemic was the constant change in protocols and healthcare measures [11]. From a qualitative study conducted during the first wave of the COVID-19 pandemic, with 36 Canadian nurses, clear and transparent communication from leadership was noted as lacking. Additionally, the study emphasized the need for psychological support to nurses [24]. It is reported that many clinicians have perceived insufficient and not appropriate preparation for providing services in-person, with some citing a lack of proper PPE and other resources [11, 25]. For example, there was tension between following health guidelines and providing care that supports the therapeutic relationship. Sometimes clinicians had to make the decision to take off their mask if it was frightening to their client, which caused a moral dilemma for many [25]. As the pandemic progresses, mental health providers will also have to address hesitancy among populations who are particularly vulnerable to vaccination fears including those who suffer from anxiety, panic attacks, obsessive-compulsive disorder [26]. As such, there is the potential for MHPs to experience work-related stress if guidance is not offered to them to help address vaccine hesitancy among their patients.

**Rationale and aims of the study.** There is an increased need to focus on the well-being of healthcare workers who are exposed to a high degree of stress and suffering and require their own professional supports [27]. Research conducted during previous pandemics primarily focused on medical health care workers, such as physicians and nurses. Existing literature has informed our understandings about the drastic negative impacts of the COVID-19 on the physical and mental health of MHPs, particularly illuminating that stressors can come from different sources within the workplace (e.g., supporting clients as well as learning to deliver remote care) [12] as well as have progressively negative effects (e.g., worsening of mental health) [7]. However, there is a paucity of evidence from MHPs who work primarily with children, leaving limited understandings of how different aspects of the workplace impact the physical and mental health of MHPs. There is some research on MHPs, however there is a paucity of evidence from MHPs who work primarily with children, with much of this research from MHPs working with an adult population outside of a hospital setting. Given the immense impact of the COVID-19 pandemic on society, and building on previous research that shows pandemics have a large impact on how health care providers deliver medical care, as well as the impact of pandemics on their professional and personal lives, it is imperative to better understand the impact of pandemics on the clinical and personal lives of MHPs working with children. The study addressed this gap by exploring the practice shifts, experience and perceived impacts of COVID-19 on pediatric MHPs during the early months of the pandemic, March -June 2020. Such learnings will enhance our understandings on different sources of stressors for MHPs as well as inform future organizational approaches and practices that promote positive mental well-being and work-life balance among MHPs.

## Methods

### Study participants and recruitment

MHPs were recruited from a public health care system. Members of the study team had conducted research during previous pandemics [28] and from this experience, it was thought that pediatric MHPs would encounter large increases in distress from the population they work with. In order to recruit this population, a purposive sampling technique was utilized [29]. This sampling method is employed to ensure that the research team is able to identify individuals who have direct, lived experience of the topic of concern [29, 30], in this instance, frontline MHPs who were able to discuss their experiences at delivering mental health support to a pediadric clientele during the early months (March-June 2020) of the pandemic [29]. With the support of the appropriate program managers, the research team approached specific mental health services to propose participation in the study. The research team initially issues invitations to eleven groups of MHPs, however, some providers from teams did not participate because of scheduling issues. These individuals were offered an opportunity to meet with a member of the research team for an individual interview; two individuals scheduled individual interviews and 5 were unable to participate due to scheduling issues. Ultimately, there were 98 participants. Inclusion criteria encompassed employment in the regional health care authority, professional provision of mental health services, and actively working through the early months (March -June 2020) of the pandemic. Potential participants were excluded if they provided primarily medical care, and if they worked with a predominantly adult population.

The research team determined to utilize focus groups as they often facilitate the generation of collective views on a subject matter [31] and are useful in eliciting rich and thoughtful understandings of participants experiences [31]. Since the beginning of the pandemic, focus group methodology has been a research tool to explore mental health provider perspectives of the COVID-19 pandemic [32–34]. The groups were familiar with each other, so the researchers anticipated some feelings of familiarity, which would further assist in elucidating comments from the participants [31]. Focus groups were scheduled with mental health programs during regular meeting times, coordinated with program leadership, ensuring that as many individuals employed by those programs could participate voluntarily. The focus groups had between four and 12 individual MHPs, with the average being seven. One individual participated in multiple focus groups, as they were a member of multiple groups.

### Data collection

Semi-structured qualitative focus groups were conducted with participating MHPs in spring and early summer 2020. Researchers obtained consent and demographic information from participants verbally, per guidelines that were approved by the University of Calgary Research Ethics Board. The duration of the focus groups was approximately 90 min and the individual interviews 45 to 60 min. Focus groups were recorded via zoom, transcribed verbatim, and all identifying information was removed.

The interview guide was adapted from previous research conducted during the SARS pandemic [28]. The previous study, following a qualitative description model, intended to provide participants with the opportunity to document their experiences with the SARS pandemic, reflect on policy and practice implications, and to discuss areas of learning that will guide future pandemics [28]; given these stated goals of the previous research, the interview guide of the current study emulated the source paper in addressing pandemic-related experiences, programming, policy and lessons learned. The two authors (DN and GD) who assisted in adapting the interview guide and conducted the focus groups are both social workers, with vast experience conducting research with MHPs and youth with mental health concerns, and both have extensive experience in qualitative interviewing and analysis. The interview guide was created to be flexible and open ended, and aimed at better understanding the impact of the pandemic on MHPs. The focus groups were not confined to written questions, and the interviewers accommodated some degree of free-flowing conversation to allow all the participating MHPs ample opportunities to reflect on the role that the pandemic has had on their lives and practice. A selection of questions asked is included in Table 1.

**Table 1:** Focus group interview guide questions

How has the pandemic affected the way you provide mental health services?
How has the pandemic affected the families/caregivers/youth you work with?
How has the pandemic affected your team and your ability to deliver mental health services?
What challenges or positive outcomes have you noticed with delivering care virtually to young people and their families?
What impact has the pandemic had on the services you are providing to youth and families?

### Data analysis

Data analysis followed the framework presented by Gale et al. [35] and Srivastava & Thomson [36]. A branch of thematic and qualitative content analysis, this framework was developed to help identify relationships within data [35]. This method of analysis was selected due to its ability to easily compare data (responses) across cases (focus groups). It is comprised of multiple steps [35, 36] including: transcription, familiarization, coding, identifying a thematic framework, indexing, charting and mapping, and interpretation [35, 36].

Recordings of focus groups were transcribed verbatim and checked for accuracy; all identifying information was removed before transcripts were sent to the analysing team of two research assistants. Following this, the first step undertaken by the analysis team was to thoroughly read the transcriptions to become familiar with the data [35]. The two research assistants then began to identify phrases (preliminary codes) to describe the content of the transcripts. For this initial coding process, the analysis focused on a small sample of the transcripts to guide the creation of descriptive phrases [35]. The research team then met to compare the initial codes identified by the research assistants, and began to formulate a preliminary codebook. The preliminary codebook comprised of descriptive phrases, definitions and examples that were approved after comparing the preliminary phrases [35]. The codebook was discussed with three team members present, allowing for any disagreements to be resolved by consensus. The codebook was agreed upon by the research team, including a co-principal investigator who has expertise in qualitative research (GD). After agreement was reached by the team on what to include in the codebook, it was applied to the remaining transcripts [35]. Following the application of the codebook to all transcripts, the research team met to interpret the coding, and organize the codes into themes in describing the data [35, 36].

Trustworthiness was ensured by multiple methods. First, the research assistants who undertook the coding worked separately from each other to ensure that they were both independently finding agreement in their processes. Secondly, the research team also met regularly throughout the process to discuss any disagreements and to establish an agreed-upon understanding of the codes. In the event of any disagreements in coding, a third team member (KB) was able to arbitrate. The research assistants also kept detailed notes and memos to reflect on the decision-making process of coding. NVivo 12 software, a data analysis and management software which allows the team members to work independently and easily compare their coding, was used during this process.

## Results

Qualitative focus group findings reflected the experiences of n = 98 MPHs delivering services to children and youth and their families throughout the early days of the COVID-19 pandemic. Demographic information (gender, mental health provider role, years of service) of the sample is summarized in Table 2. The majority of participants were female (n = 82), and most of the participants had been employed in this field between 5 and 20 years. Participants worked in a wide range of professions in pediatric mental health, including roles of social worker, counsellor, psychiatrist, nurse, supervisor and various alternative mental health roles. Due to the large number of professional role designations, all participants are referred to MHPs hereafter.

**Table 2:** Demographic Information of Participants

**Position title**	
Social Worker	12
Health Care Management	10
Mental Health Care Provider (therapists, counsellors, etc.)	31
Psychologist/Psychiatrist	16
Medical Care Provider (Doctor, nutritionists, physical care providers, etc.)	12
Registered Nurse	7
Misc.	10
**Length of time employed in health care system**	
Less than 5 years	13
5–10 years	28
11–20 years	38
More than 20 years	12
Unknown	7
**Gender identity**	
Male	14
Female	82
Did not identify	2

Participants noted that providing mental health services during the early months of the COVID 19 pandemic was an unprecedented and ‘incomparable’ experience, with many struggling in experiencing the pandemic alongside the youth and their families with whom they worked. As one provider highlighted succinctly: “*This is likely the first time that we as therapists are experiencing the same trauma or the same type of – I guess you can call it incident trauma whatever – as our clients are, and so we are trying to figure out how to navigate this while at the same time hold space and support not only [for] our staff but also our clients (FG9).”* Three major themes developed from inductively analyzing the transcripts. First, MHPs reflected on what it was like to rapidly transition to virtual care and working from home to comply with public safety measures. Second, many providers conversely discussed their complicated feelings about working onsite during the pandemic and the mental health toll and stress of working outside of the home. Finally, while discussing their shift from in-person to virtual care, many MHPs experienced high levels of stress, exhaustion and burnout at work, with diminished boundaries between their personal and work lives.

### Shift to virtual delivery and work from home

With the outbreak of the COVID-19 pandemic in March 2020, the mode of delivering mental health services moved rapidly from in person to virtual care due to new public health guidelines. As demonstrated by one provider: “*Everything’s online, everything. The whole like medium of service delivery has completely changed and it changed within two weeks* (FG2).” Many MHPs reported experiencing increased demands, and needed to concisely communicate changes in service delivery to their clients and reassure them that services would continue. As one participant noted: “*I just remember sort of being both physically and like mentally exhausted … the anticipation of like potentially disappointing families that we couldn’t provide the service that they had hoped to gain from us or were gaining from us (FG6).”* The pressure to inform clients to the shift in delivery, without knowing precisely for how long this type of care would be delivered was equalled by the pressure to quickly learn new modes of delivering mental health services. Some MHPs also felt that the shift in service modality lessened the perceived efficacy of their clinical work, as one noted: “*In terms of the staff, a theme that has come up a lot is that they have been feeling just not very effective … (FG9).”* Many providers stressed that they were concerned that they were not able to serve their client’s as effectively as prior to the pandemic, while balancing multiple changes and adapting their practice to providing services virtually.

Providers found that shifting to virtual delivery and work from home decreased variety in their days. Many saw an increase in monotonous computer work and highlighted that their focus and attention were diminished due to the newfound repetitive nature of their work. Some providers noted that previously their work had been mobile and provided diverse daily opportunities, however all this changed rapidly and their days now consisted of hours of computer time. As one provider elaborated, in the past: “*our job used to be so diverse. Sometimes we were on the computer when we were report writing, sometimes we were in the office, sometimes we were out in the sunshine, even if you were just driving to a visit… now everything is on a screen for so much more of our day than it used to be. It didn’t used to be actually 8 hours a day (FG5).”* Spending increased amounts of time at the computer appeared to diminish some providers feelings of satisfaction with their work.

MHPs noted that while working from home they felt less connection to co-workers and clients, as highlighted by one provider: “*it’s like, oh it’s COVID, oh, you can’t see anybody. So I felt like I was completely cut off from, not only my clients that I only just had a few in person sessions with, but also the whole team (FG11).”* One provider succinctly highlighted the increased feeling of isolation that many other MHPs touched on: “*I mean at home you always feel a little bit more isolated from the team, which is the biggest part I guess I miss. You’re able to just go over to somebody else’s office and consult with them about a case or you know, if you can kind of see somebody’s having a rough day, you can kind of have a chat and hopefully help out a little bit, so that part’s missing (FG6).”* The conversational and social aspect of the workplace diminished as individuals working from home were unable to have easy conversations with their co-workers. There was also a growing disconnection from supervisors, as MHPs noted fewer check-ins with leaders who, in some cases, had become responsible for addressing pandemic system-level issues. Further, the lack of ability to check in and have quick and casual conversations with other staff members left many providers feeling isolated and disconnected.

While many MHPs felt overwhelmed by the sudden changes to their mode of delivery, there were some that noted many positives in the shift. Many MHPs were able to adapt quickly to the changes and saw that shifts in their mode of delivery allowed for greater flexibility and increased likelihood of positive changes being accepted into the provincial health system, as one MHP highlighted: “…*it’s been really positive to see how as health care providers how quickly we have changed, how quickly we have adapted, how quickly in the past if you wanted to put forward a policy or change in practice it took years to do (FG4).”* Other MHPs also found that the shift to working from home allowed them to have extra flexibility within their schedules and more time due to eliminating their commute: *“not being stuck in my car for 30, 40% of the day driving between sessions sometimes, created a lot more flexibility in my schedule. So that has been nice (FG11).”* Mental health supervisors found positive changes among their staff, noting that MHPs showed increasing vulnerability and willingness to ask salient questions. Ultimately, many MHPs reported being able to continue performing their job to quite a high level, and were able to ensure that their clients could receive the help needed during an extremely tough time: “*I think we’re as resilient as we could have been, and we maintained our professionalism, and we kept the clinic going as best that we could, and I’m very proud of the fact that we all just still manage that (FG10).”* With the many substantial changes in their ways of practicing and offering mental health services, some MHPs noted that they were well suited for shifting to work from home; however overall, the lack of diversity in their daily duties wore on them over time.

### Concerns about working in-person

While working from home brought very unique challenges to MHPs, working in person on-site also raised substantial concerns. For some, there was perceived to be less concern given by leadership to personal and family circumstances that might make in-person work uncomfortable, as highlighted poignantly by a MHP: “*I have to say that at the beginning, it felt very uncomfortable that our first priority was around, you’ve got to be in at work, we have to still continue as a service, whereas out there, everybody’s taking all these safety measures and we weren’t… what makes me upset about some of that too, it puts me at risk, puts my family at risk, puts my parents at risk, because I am in the sandwich generation (FG5).”* While MHPs wanted to offer services similar that prior to the pandemic, MHPs who had to work in-person were more anxious that they might bring the virus home to family members, many of whom were considered vulnerable. While most expressed frustration about working in person, especially during the early months of the pandemic when it seemed that most other mental health services had shut down, some clinicians preferred working on site, expressing that working from home did not allow them to have distance between their work and they felt much more effective when physically in the office.

### Exhaustion and stress of working through the pandemic

Following multiple changes to service delivery, MHPs shared experiencing extreme levels of stress. MHPs who were working from home spoke about the competing needs between their families and their clients, and their increased levels of burden to provide adequate time and care to both. As one provider noted, the distinction between personal and professional lives was becoming blurred: “*I find that the struggle for me is… before I could… more easily separate the professional and personal roles in my life. And now… I find a lot of times we’re in both at the same time, and it’s just really difficult (FG2).”* Further, because schools were closed for in-person learning, MHPs with school-age children were also in charge of supporting and monitoring their children in their schooling. Some MHPs noted struggling with being able to give their clients their full attention while their children also required care, with one highlighting: “*As parents we have children at home who are online schooling, and we’re struggling with that, but yet we have to be “on” and present in a well sort of packaged way for our clients who are experiencing the same thing (FG9).”* MHPs found pressure to successfully support their children with online learning while still maintaining a full client load and providing the same level of care, leading to feelings of ineffectiveness. Being clinically responsible for their clients’ care while struggling to support their families left MHPs working longer hours in order to ensure that their clinical load was being handled and that they still had enough time to devote to their families.

Living through the pandemic *and* providing quality care to their clients, caused emotional and physical exhaustion for many providers. One clinician aptly stated this phenomenon, as follows: “*it’s work work work and then for me, when I’m not working, I’m just exhausted – far more exhausted than I normally am…but there’s an exhaustion that I’ve never experienced before…. I think it’s emotional exhaustion (FG7).”* Others expressed similar sentiments, noting that they felt like they were always on high alert to support their clients while remaining acutely attuned to the needs of their families, and there was minimal downtime because there was no travel or break time between meetings. MHPs felt they were increasingly becoming at risk for burnout and worried about the longevity of their ability to work at this pace. One MHP noted: *“I do wonder if that’s part of the burnout… Now there’s almost less of that downtime because now, 100% of the time is client directed. (FG3).”* Many MHPs noted that they were emotionally exhausted from their continued service provision, with one succinctly noting: “*I’m exhausted…And I think the balance of balancing your own life, holding emotional space for other people, home schooling, the whole gamut is hard for us (FG5).”* MHPs reported that the combination of isolation from their colleagues, limited opportunity for breaks in the day, and increased personal demands on their time to care for their own families, was leading to increased levels of personal and professional exhaustion.

As illustrated in these findings, the COVID-19 pandemic created uncertainty for MHPs, with many changes to the service delivery mode, blurring of boundaries between personal and professional lives, and physical and emotional exhaustion of working double and sometimes triple duty. They felt a strong sense of ethical and moral responsibility to maintain a high quality of care for their clients, while also struggling to care for their children and other members of their families. Despite these pressures and challenges, many MHPs perceived themselves and their colleagues as resilient in finding creative and supportive ways to collaborate, communicate and support one other, as summed up by one MHP: “*I just feel so lucky to work with the team that I do because we are a team that [is] very cohesive, very innovative team. We really work well together, we have good professional relationships, and so that has definitely made the work easier. And I felt supported in that way because we’re as a program ‘all in the same boat’ trying to navigate this challenging scenario for our patients…(I1).”*

## Discussion

The present study sheds light on the impact of the COVID-19 pandemic on pediatric MHPs, and guides us towards better preparedness for the future. This study identified reasons for heightened emotional stress, anxiety, fear and depression among frontline clinicians during the early phase of the pandemic. Causes of these challenges comprise the rapid transition of in-person to virtual care, fear of working in the office and away from home, feeling unsafe, the need to comply with ever-changing public safety measures, and difficulty striking a work and life balance – some of these challenges and causes for anxiety coincide with results in other studies [8, 11, 12].

The emerging virtual work modality required during COVID-19 added immense pressure and uncertainty in MHPs’ daily lives. The new routine became monotonous for some; they lost the ‘personal touch’ and relational connection previously common in their work, and felt isolated, unsupervised and under-supported. Many were not interacting with other staff members, even for consultation, making work more stressful at professional and personal levels.

MHPs’ stress and exhaustion call for immediate proactive thought and action. At the early stage of the COVID-19 pandemic, MHPs were anxious and fearful. They were apprehensive of disappointing families in need of mental health services by not meeting their care delivery expectations as they shifted the service mode from in-person to online. The MHPs generally expressed challenge in how to communicate changes in service modality to clients which added challenge and stress. Many MHPs seemingly lacked confidence and sufficient virtual care knowledge to be as effective as they sought to be. This presents opportunity for learning for supervisors and managers in bettering supporting MHPs with guidance, training and timely consultation.

Beyond the many challenges faced by MHPs in the pandemic, this study also highlights positive outcomes emerging from a new work routine in the pandemic. Further, MHPs recognized their versatility in quickly adopting to required changes responding to new ways of efficiently working [37]. The clinicians in this study continued working in their roles even within the new working environment and they demonstrated resilience and professionalism in their practice.

### Limitations and future directions

Using purposive sampling to identify the most appropriate individuals for the study, 98 participants took part in the research, of which 82 of them were women. To best understand the impact of the COVID-19 pandemic on the psycho-social wellbeing of MHPs, the inclusion criteria should more equitably cover sex/gender variation. This research further invites study examining new solutions, which warrants critical reflection on the fact that the present study was based largely on female MHPs’ responses, thus rendering male views in the minority. Additionally, while attempts were made to ensure that all disciplines of mental health providers were included, given the wide variety, we were unable to achieve full representation. In future studies, it would be prudent to explore perceptions from additional disciplines.

This research informs future studies. For instance, it has been over two years since the pandemic began, and many shifts have occurred in the workplace and throughout society in general. Initially, social distancing and lockdowns were put into place, and now masks have gradually become part of our lives and/or no longer required. The nature of work to remote and virtual care, has now shifted to variably hybrid and/or many MHPs have returned to previous in-person work locations. Our findings focus on the early phase of the pandemic, and thus need to be updated as shifts have ensued and continue to evolve, and MPHs continue to work in strained contexts and environments. Over time, experiences working with technology-based modalities in the workplace likely have changed. Future studies can analyse the paradigm shifts during different phases of COVID-19 and what mechanisms or interventions have supported the practitioners to cope with various changes and continue to serve their clients effectively.

Future research can substantiate our understanding of the nature of the support structure required by MHPs during such challenging times. This study and others [11, 37] have touched the surface about possible human resource requirements, such as the need for enhanced skills and competency supports in dealing with clients during such unprecedented times [27, 37]. Additionally, our study begins to suggest that there were positive experiences of working within teams and the efficacy of said teams could be examined further, particularly in regards to working throughout times of extreme stress. Practical supervision skills to guide and support MHPs in a timely manner, effective feedback mechanisms, and training of staff in various areas such as self-regulation and self-care are needed, particularly to buffer burnout and stress, and support well-being.

## Conclusion

Similar to the experiences of other frontline service providers in health care, pediatric MHPs faced the immense impact of COVID-19. This study has amplified MHPs’ experiences and reasons behind these experiences. Additionally, the study has offered direction for needed supports and structures in pandemic circumstances, with relevance currently and in the future.

Despite being negatively affected by the pandemic, MHPs have shown resilience, vulnerability, and courage in navigating the transition from in-person to online practice amidst themselves living with and navigating the pandemic. The research importantly has shared the voices of MHPs and has thus posed some critical questions for further planning and research. MHPs need additional support from their supervisors and the system to continue to provide care and ensure their own well-being in pandemic contexts.

## Electronic supplementary material

Below is the link to the electronic supplementary material.

Supplementary Material 1

## Data Availability

Available from the corresponding author on reasonable request.
