# Supplementary material for: Examining the experiences of pediatric mental health care providers during the early stage of the COVID-19 pandemic
Source: BMC Psychol. 2023 May 4;11:147. doi: 10.1186/s40359-023-01170-x (PMC10157584; doi:10.1186/s40359-023-01170-x)
Supplement: Supplementary file 1 — Supplementary Material 1 [file 40359_2023_1170_MOESM1_ESM.docx]

COREQ Table 1

| Consolidated criteria for reporting qualitative research—a 32 item checklist for interviews and focus groups | | |
| --- | --- | --- |
| Number | Item | Guide Questions/Description |
| **Domain 1: Research teams and reflexivity** |  |  |
| Personal Characteristics |  |  |
| 1 | Interviewer/facilitator | Which authors conducted the interviews? |
|  |  | DN, GD conducted the interviews |
| 2 | Credentials | What were the researcher's credentials? |
|  |  | GD and DN both have PhD |
| 3 | Occupation | What was their occupation at the time of study? |
|  |  | Associate Professor (GD), Associate Dean (DN) |
| 4 | Gender | Was the researcher male or female? |
|  |  | GD: Female, DN: Male |
| 5 | Experience and training | What experience or training did the researcher have? |
|  |  | Both researchers have training and extensive experience in qualitative interviewing |
| Relationships with participants |  |  |
| 6 | Relationship established | Was a relationship established prior to study commencement? |
|  |  | Participants were recruited from AHS, and many were already familiar with the researchers through other research projects |
| 7 | Participant knowledge of the interviewer | What did the participants know about the researcher? I.e. personal goals, reasons for doing the research |
|  |  | Some of the participants were familiar with the researchers through other projects, and are aware of their fields of interest and experience |
| 8 | Interviewer characteristics | what characteristics were reported about the interviewer/facilitator? |
|  |  | No biases/assumptions were reported. Participants may have been informed of educational background of interviewers, which may have helped clarify why they were interested |
| **Domain 2: Study Design** |  |  |
| Theoretical framework |  |  |
| 9 | Methodological orientation and theory | What methodological orientation was stated to underpin the study? |
|  |  | Fundamental qualitative description, framework method |
| Participant Selection |  |  |
| 10 | Sampling | How were participants selected? |
|  |  | Purposive sampling |
| 11 | Method of approach | How were participants approached? |
|  |  | Participants were recruited through their involvement in AHS youth mental health/youth health |
| 12 | Sample Size | How many participants were in the study? |
|  |  | 98 |
| 13 | Non-Participation | How many people refused to participate or dropped out? Reasons? |
|  |  | No reasons were given for not participating and no one dropped out after recruitment commenced |
| Setting |  |  |
| 14 | Setting of data collection | Where was the data collected? |
|  |  | Data was collected and recorded via zoom |
| 15 | Presence of non-participants | was any else present besides the participants and researchers? |
|  |  | An additional research assistant was present during the focus groups to make notes |
| 16 | Description of sample | what are the important characteristics of the sample? |
|  |  | 98 participants were recruited, 82 were women, 14 were men 2 did not identify, majority had a long tenure within the health care system (5-20 years) |
| Data collection |  |  |
| 17 | Interview guide | Were questions, prompts, guides provided by the authors? Was it pilot tested? |
|  |  | Interview guide was not provided to the participants ahead of time, it was not pilot tested |
| 18 | Repeat interviews | Were repeat interviews carried out? If yes, how many? |
|  |  | No repeat/follow up interviews were conducted, but one participant participated in 2 focus groups |
| 19 | Audio/Visual recording | Did the research use audio or visual recording to collect the data? |
|  |  | all interviews were audio recorded via zoom |
| 20 | Field notes | Were field notes made during and/or after the interview or focus group? |
|  |  | Notes were made regarding demographic information during the focus groups, but no additional field notes were made |
| 21 | Duration | What was the duration of the interviews or focus groups? |
|  |  | All focus groups were between 45-60 minutes |
| 22 | Data Saturation | Was data saturation discussed? |
|  |  | Saturation was discussed |
| 23 | Transcripts returned | Were transcripts returned to participants for comment and/or correction? |
|  |  | No, transcripts were not returned to participants |
| **Domain 3: Analysis and findings** |  |  |
| Data analysis |  |  |
| 24 | Number of data coders | How many data coders coded the data? |
|  |  | 2 coders analysed the data |
| 25 | Description of the coding tree? | Did authors provide a description of the coding tree? |
|  |  | The coders worked inductively to create a codebook |
| 26 | Derivation of themes | Were themes identified in advance or derived from the data? |
|  |  | Themes were identified from the data, they were not identified in advance |
| 27 | Software | What software. If applicable, was used to manage the data? |
|  |  | Nvivo 12 was used |
| 28 | Participant checking | Did participants provide feedback on the findings? |
|  |  | Findings were not presented to participants for feedback |
| Reporting |  |  |
| 29 | Quotations presented | Were participant quotations presented to illustrate the themes/findings? Was each quotation identified? |
|  |  | Participant quotations are provided in the results to illustrate the themes/findings. As all data was collected during focus groups, it was not possible to individually identify participants so all quotes were identified by their focus group number |
| 30 | Data and findings consistent | Was there consistency between the data presented and the findings? |
|  |  | Yes, there is consistency between the data and the findings, with the data presented being illustrated by direct quotes from participants |
| 31 | Clarity of major themes | Were major themes clearly presented in the findings? |
|  |  | Major themes are presented in the results section |
| 32 | Clarity of minor themes | Is there a description of diverse cases or discussion of minor themes? |
|  |  | There are some diverse cases mentioned in the results and discussion section |
|  |  |  |
